# Sensing, monitoring, and release of therapeutics: the translational journey of next generation bandages

**DOI:** 10.1117/1.JBO.24.2.021201

**Published:** 2018-12-27

**Authors:** Zongxi Li, Haley Marks, Conor L. Evans, Gabriela Apiou-Sbirlea

**Affiliations:** a Mass General Research Institute, Boston, Massachusetts, United States; b Massachusetts General Hospital, Harvard Medical School, Wellman Center for Photomedicine, Charlestown, Massachusetts, United States

**Keywords:** translational research, sensing, monitoring, and release of therapeutics bandage, phosphorescence imaging, oxygen-sensing metalloporphyrin

## Abstract

This article aims to be a progress report on the Sensing, Monitoring And Release of Therapeutics (SMART) bandage—one of the three technologies that received the inaugural SPIE Photonics West Translational Research Symposium Award in 2015. Invented and developed by Dr. Conor L. Evans and his research team at the Wellman Center for Photomedicine, Massachusetts General Hospital, the SMART bandage is a tool aiming to provide measurements of physiological parameters in the skin alongside the administration of therapeutics on-demand. Since the project began in 2012, the chemists, physicists, and biomedical engineers in the team have worked closely with partners from academia and industry to develop oxygen-sensing SMART bandage prototypes that are now in first-in-human clinical studies. This report gives perspectives on the genesis and translational journey of the technology with an emphasis on the challenges encountered, and the solutions innovated at each stage of development.

## 1 Introduction

In 2012, Dr. Conor L. Evans attended a meeting at the US Army Institute of Surgical Research that fundamentally changed his team’s research priorities. After meeting wounded warriors and the physicians, nurses, and caregivers who guide them back to health, Dr. Evans and his team were inspired to create new bandage technologies that could both provide direct measurements of wound physiology while simultaneously promoting wound healing.

This sensing, monitoring, and release of therapeutics (SMART) bandage was designed with the potential to modularly include (1) a sensing component that responds to changes in various relevant physiological parameters (e.g., oxygen concentration, pH, bacteria load, etc.), (2) an optical readout device that provides real-time monitoring of these parameters via a sensor or camera, and (3) a drug release component that allows for the on-demand delivery of therapeutics (anti-inflammatories, analgesics, antibiotics, etc.) for intervention.

The goal of the SMART bandage, once developed and validated, was to provide a “window into the wound” that could be used in combination with existing hospital systems as well as personal connected devices, such as smartphones. This approach would allow the status of the wound to be easily monitored by a caregiver, who may in turn trigger the release of therapeutics for interventional purposes.^1^^–^^4^

**Fig. 1:**
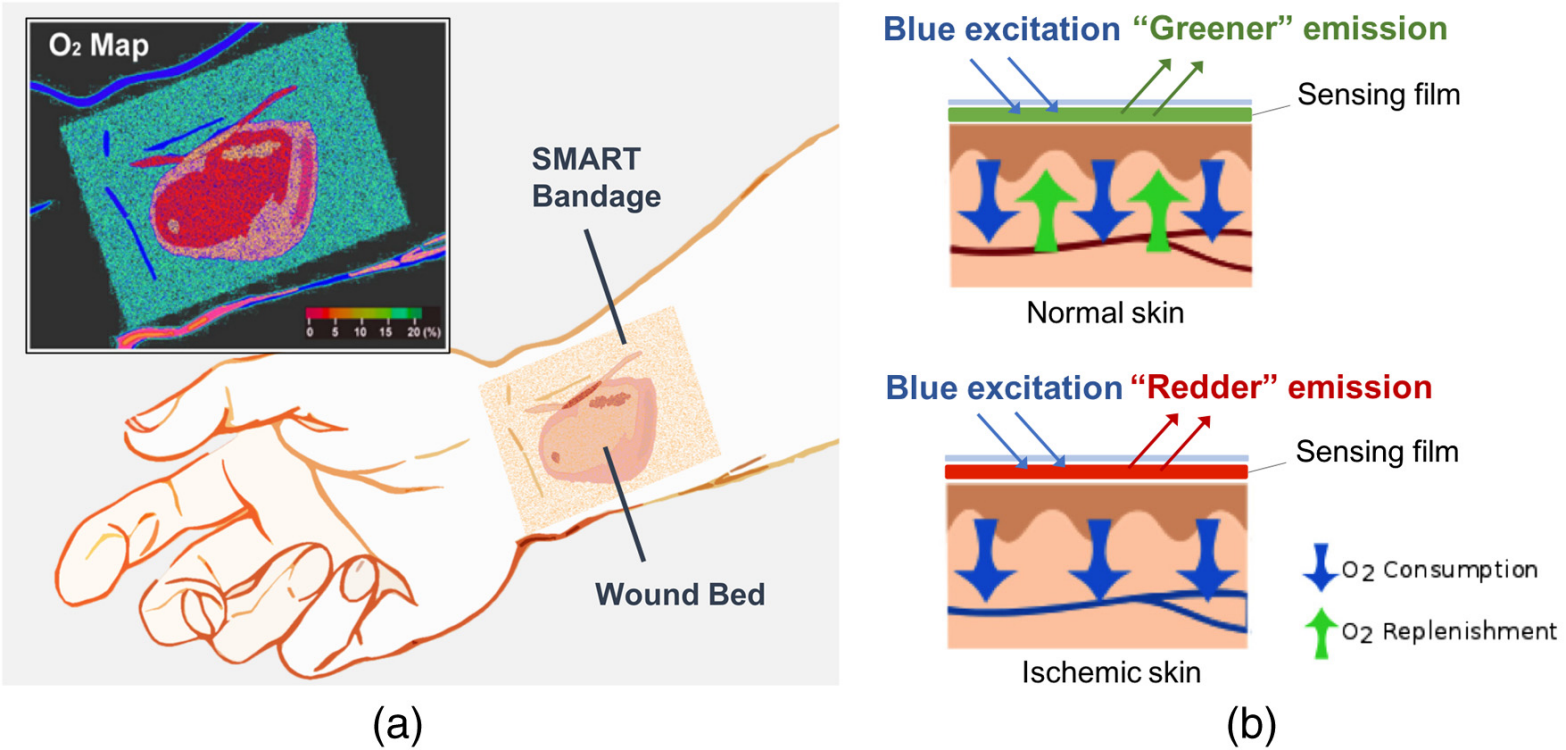
(a) Schematic illustration of the oxygen-sensing SMART bandage. (b) Top: oxygen-sensing SMART bandage applied to normal skin. The oxygen concentration in the tissue is high, quenching the red emission of the sensing phosphor. The film emits green color overall from the fluorescent reference dye. Bottom: oxygen-sensing SMART bandage applied to ischemic skin. The oxygen concentration in the tissue is low, the strong red emission of the sensing phosphor overcomes the green fluorescence of the reference dye. The bandage emits red color overall.

Between 2012 and 2016, SMART bandage prototypes were developed for the Department of Defense’s high priority physiological parameter: tissue oxygenation. Initial development focused on solving the problem of measuring tissue oxygenation transdermally for situations including burn wounds, skin grafts, and tissue transplants [Fig. 1(a)]. Tissue oxygenation is an important parameter used to clinically predict whether a wound will heal properly, or if a skin graft/flap will be successfully integrated after reconstructive surgeries.^5^^,^^6^ Existing tools often suffer from several limitations. For example, many oxygen-sensing clinical tools require complex and bulky equipment, perform indirect measurements, provide single point measurements, or require dressing removal that can cause wound disruption and infection. In discussions, plastic surgeons and wound care physicians consistently voiced the need for noninvasive, easy-to-use tools that could map skin oxygenation across a wound and its surrounding skin area. To specifically provide a solution to address this unmet clinical need, the oxygen-sensing SMART bandage was designed to be composed of a biocompatible sensing film and an imaging readout device.

The sensing film was engineered to contain a phosphorescent metalloporphyrin whose red emission was modulated by molecular oxygen^7^ and a fluorescent reference dye that provided an oxygen-insensitive green emission [Fig. 1(b)]. Using the ratio between the red and green emission intensities enabled the accurate calibration of the bandage’s emission to oxygen concentration using a function known as Stern–Volmer equation:

$$I_{0}/I=\tau_{0}/\tau =1+K_{sv}\cdot {\mathrm{pO}}_{2},$$

where I and τ are the phosphorescence intensity and lifetime at oxygen concentration ${\mathrm{pO}}_{2}$ and in the absence of oxygen $\left(I_{0},\tau_{0}\right)$, and $K_{SV}$ is the Stern–Volmer quenching constant. This relationship describes an important aspect of the material’s performance: the sensor responds with increased red emission as oxygen concentration decreases. As low oxygenation is typically associated with poor tissue health, this material response is well tuned for the detection of pathogenic states.

The readout device was initially configured as a customized camera with red and green filters to capture the phosphorescence from the sensing film. At the time the project received the SPIE Translational Research Award in February 2015, the SMART bandage prototype had already been validated *in vitro* and *in vivo* in preclinical studies of porcine and rat models for burns, grafts, and ischemic injuries.^8^

After considerable preparation, the next major developmental step occurred in the spring of 2017: the SMART bandage was approved by the Beth Israel Deaconess Medical Center (BIDMC) Institutional Review Board (IRB) for a first-in-human clinical study under the guidance of Dr. Samuel Lin. This first human study was focused on deep perforator flap transplants in the context of breast reconstruction surgeries post-mastectomy. By the fall of 2017, two additional clinical trials were approved at Massachusetts General Hospital (MGH) in collaboration with groups affiliated with the Wellman Center for Photomedicine. The first, led by Drs. Conor Evans and Yakir Levin, was designed to use the SMART bandage to detect effects of chronic ultraviolet light exposure on skin oxygenation and oxygen consumption in healthy volunteers. The second, led by Drs. Daniela Kroshinsky and Adam Raff, leveraged the SMART bandage to detect inflammation as a potential marker to diagnose the skin disease known as cellulitis.

In January 2018, due to the efforts of Dr. Haley Marks in the Evans lab, the project received the SPIE “Franz Hillenkamp Award” to further develop the SMART bandage technology to be used for optically mediated drug release in exudative wounds. As of the summer of 2018, prototypes under development incorporate customized oxygen-sensing phosphors into a drug-releasing polymer matrix to allow release of therapeutic molecules on demand. Additionally, several new oxygen sensing prototypes are under development, including a wearable all-in-one device capable of monitoring, recording, and transmitting the tissue oxygen concentration and blood oxygen saturation data.

## 2 Translational Journey

**Fig. 2:**
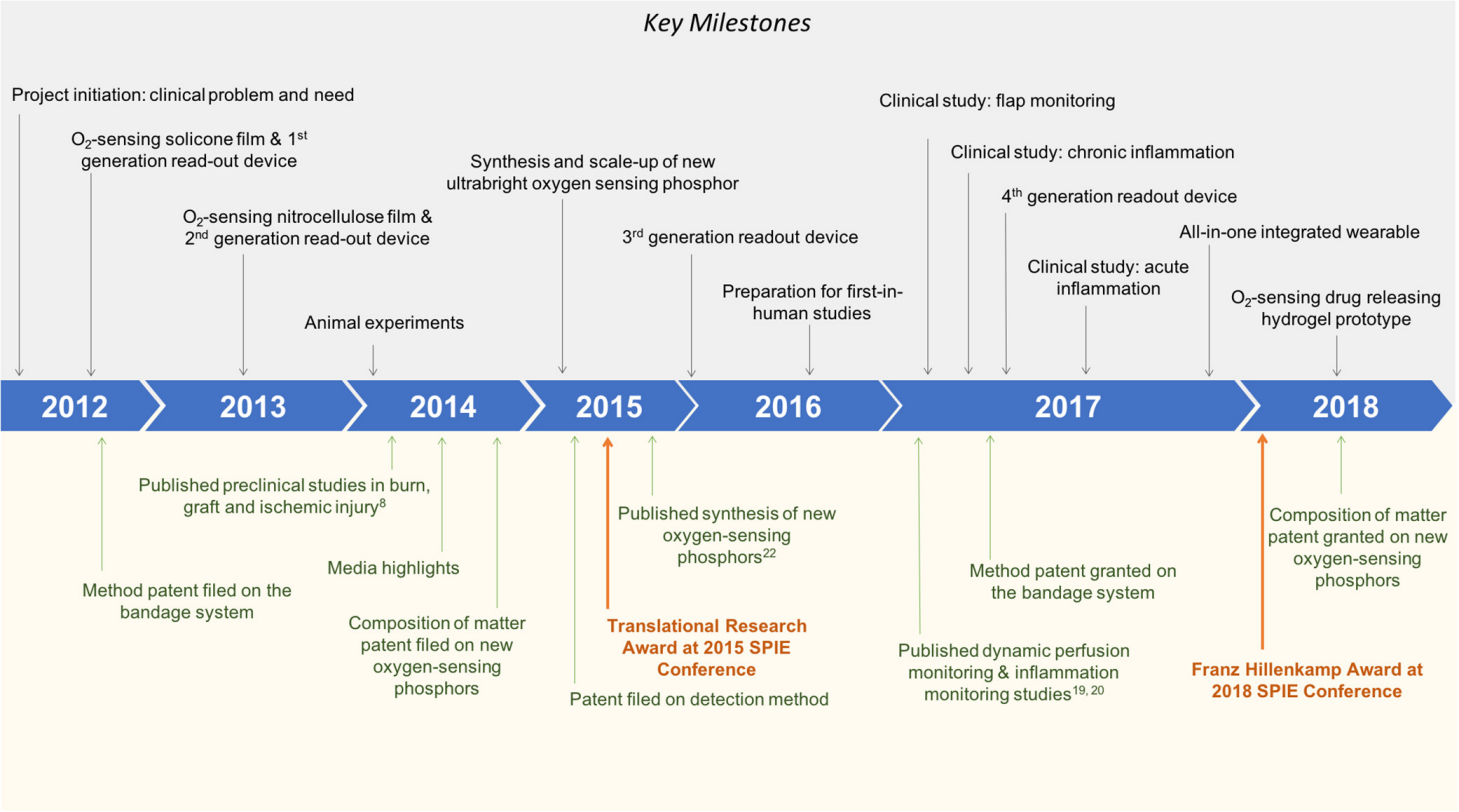
Key milestones throughout the oxygen-sensing SMART bandage translational journey. The top half of Fig. 2 summarizes the thirteen milestones, while the bottom half of the figure shows the most relevant publications, new intellectual property, and awards.

This section provides a chronological review of the SMART bandage translational research stages, and is organized under 13 subsections, each of them representing the key scientific, engineering and clinical milestones achieved during the development process, and across the three components of the technology: (1) oxygen sensing phosphor, (2) sensing film, and (3) readout device. Challenges, such as the brightness and large-scale synthesis of the oxygen sensing phosphor, breathability of the sensing film, ease-of-use of the readout device, are described and solutions to overcome them are presented (Fig. 2).

### 2.1 Clinical Problem and Need (2012)

The SMART bandage project began with Dr. Evans’ visit to the US Army Institute for Surgical Research (USAISR), in San Antonio, Texas where Dr. Rodney Chan, a plastic and reconstructive surgeon, and his colleagues perform complex surgeries to rebuild the hands, feet, limbs, and faces of wounded soldiers. During these surgical interventions, tissues are moved to rebuild and regain function. Graft failure can occur for a number of reasons, many of which act to disrupt the oxygen supply to the transplanted tissue causing tissue death and graft failure. As autografted tissue is extremely precious and in a limited supply, there is an immense need for consistent monitoring and rapid assessment of the graft to prevent potential failure.

**Fig. 3:**
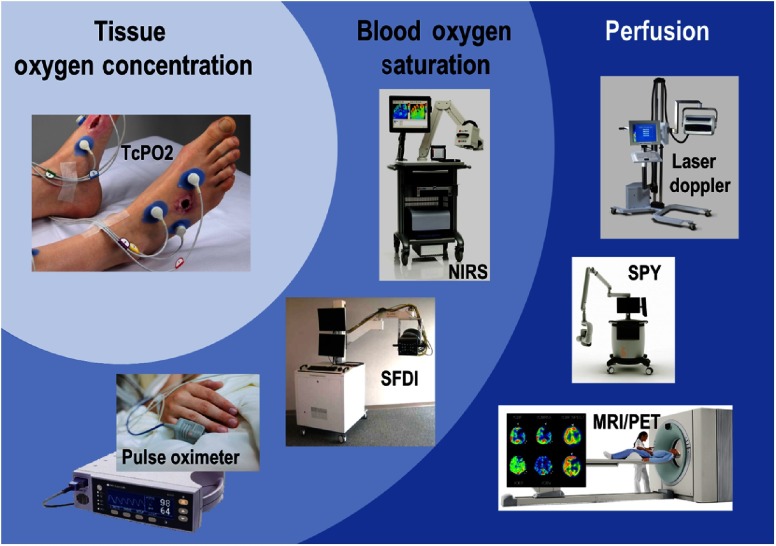
Competitive landscape for the oxygen-sensing SMART bandage. Left: tissue oxygen concentration (${\mathrm{pO}}_{2}$) measurement techniques: transcutaneous ${\mathrm{pO}}_{2}$ measurement (${\mathrm{TcPO}}_{2}$). Middle: blood oxygen saturation (% ${\mathrm{StO}}_{2}$) measurement techniques: near-infrared spectroscopy (NIRS), spatial frequency domain imaging (SFDI), pulse oximetry. Right: perfusion measurement techniques: laser Doppler flowmetry, indocyanine green (ICG) fluorescence angiography (SPY), techniques based on magnetic resonance imaging (MRI), or positron emission tomography (PET).

Many technologies working to address these issues have been brought to market and are already used in clinical practice to assess tissue oxygenation. Figure 3 classifies several examples of these tools into three categories: (1) perfusion (blood flow measurement), (2) blood oxygen saturation (oxy/deoxy hemoglobin ratio or % ${\mathrm{StO}}_{2}$), and (3) tissue oxygen concentration (oxygen partial pressure or ${\mathrm{pO}}_{2}$).

Perfusion measurement tools require specialty equipment that are often expensive, bulky, and may require injection of exogenous contrast agents.^9^^–^^12^ For example, the SPY device requires the injection of indocyanine green to visualize tissue perfusion.^10^ Blood oxygen saturation methods advantageously do not require injection of contrast agents; however as they measure the ratio of oxy- to deoxyhemoglobin, these tools do not provide a direct measure of tissue oxygen concentration.^13^^–^^15^ Tissue oxygen concentration tools such as transcutaneous oxygen monitoring (${\mathrm{TcPO}}_{2}$) do provide a direct measure of tissue oxygen content;^16^ however, these methods only perform measurements at a single spatial point, they require extensive bedside calibration for each location, and require the removal of the wound dressings placed over the surgical site, potentially causing disruption, and risking infection.^17^

The team initiated the development of the SMART bandage specifically as a tool that would (1) add to, instead of interfering with, current post-surgical workflow, (2) be user-friendly, allowing anyone—including minimally trained caregivers—to assess the health of transplanted tissue, (3) provide direct two-dimensional (2-D) imaging of oxygenation concentration, (4) allow normal visual inspection of the tissue, and (5) enable interventions such as drug release—all without changing a bandage or disrupting the graft site.

### 2.2 $O_{2}$-Sensing Silicone Film and First-Generation Readout Device (2012)

The first SMART bandage prototype developed by the team included two components: a sensing film and an imaging device. As a first test, the sensing film was composed of the commercial-off-the-shelf oxygen-sensing metalloporphyrin Oxyphor R2 physically embedded within polydimethylsiloxane (PDMS), an FDA-approved solid silicone material. PDMS was a natural first choice at this stage to minimize the potential safety and regulatory hurdles: it is polymer with well-understood physical properties used in numerous medical devices.

Interestingly, these initial studies determined that the sensor molecules tended to aggregate as the PDMS material cured, resulting in “quenching” of the phosphor emission. To address this challenge and create a sensing layer with homogeneously dispersed phosphorescence sensors, the team modified the surface functional groups on the sensor molecules and introduced cosolvents that acted to keep the sensors solvated during the curing process.

**Fig. 4:**
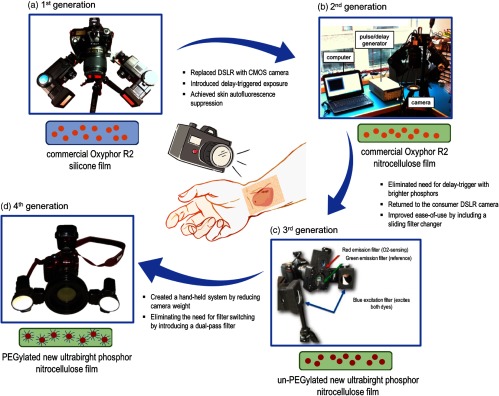
Evolution of the oxygen-sensing SMART bandage. (a) First-generation system: commercial Nikon D70 DSLR camera equipped with two regular flash units; silicone film containing commercial Oxyphor R2. (b) Second-generation system: industrial Thorlabs CMOS camera connected to an accurate delay pulse generator and a laptop computer; nitrocellulose film containing commercial Oxyphor R2. (c) Third-generation system: customized commercial Nikon D70s camera with separated red and green passing filters mounted on a sliding filter changer; nitrocellulose film containing un-PEGylated new ultrabright phosphor. (d) Fourth-generation system: “off-the-shelf” commercial Nikon D3400 camera equipped with a simple longpass filter; nitrocellulose film containing PEGylated new ultrabright phosphor.

The first-generation readout device was comprised of a simple Nikon D70 DSLR camera customized via the removal of the IR filter [Fig. 4(a)]. Two standard Vivitar flash units were mounted on the camera base and outfitted with custom-ordered 385-nm centered, 70-nm bandwidth blue excitation filters. The *in vitro* calibration of the bandage was successful and demonstrated for the first time that the emission intensity of the silicone film was correlated to oxygen concentration within the physiologically relevant range (0 to 160 mmHg). Figure 4 shows the evolution of the sensing film and readout device during the entire SMART bandage development process to date, and will be referred to throughout the rest of this section.

### 2.3 $O_{2}$-Sensing Nitrocellulose Film and Second-Generation Readout Device (2013)

Although the PDMS silicone film matrix was formulated to be highly breathable, it became obvious in initial testing that the bandage’s mechanical properties needed to be modified to form an air-tight adhesive seal with the skin. After testing potential candidate materials, the team selected an FDA approved liquid bandage, a rapid-drying nitrocellulose material that when applied to skin solidified into a conformal gas-permeable bandage. Though the breathability of the liquid bandage was advantageous for tissue sensing, the oxygen levels in the ambient air could influence the green/red emission ratio. Completely occluding gas exchange from the air was not a valid option, as skin does exchange oxygen with air. Instead, the team chose the partially permeable, FDA-approved clear film known as Tegaderm. As an added benefit, Tegaderm was found to bind tightly to the liquid bandage, which made the removal of the sensing film following use extremely easy.

When tested *ex vivo* on porcine skin, it was found that the weak phosphorescence emission of this Oxyphor R2-based bandage could be overwhelmed by the endogenous autofluorescence of the skin. To improve this weak signal-to-noise, a second-generation imaging device was built to take advantage of the bandage long-lived phosphorescence. Using a short flash to excite the porphyrin and time-gating a camera acquisition window to reject fluorescence, the team constructed a system that could specifically isolate the oxygen-sensitive phosphorescence emission. This system, which employed a Thorlabs complementary metal-oxide-semiconductor (CMOS) camera, made use of a precise pulse delay generator to introduce a reproducible time gate between the excitation light pulse and camera detection [Fig. 4(b)].

### 2.4 Animal Experiments (2014–2017)

Using the liquid bandage prototype and the second-generation imaging device, the team performed first *ex vivo* studies on a burn wound created on porcine skin explants. The data clearly showed that, as compared with Clark electrode, a well-established ${\mathrm{pO}}_{2}$ measurement technique, the bandage was able to detect the decreased oxygen consumption rate of the necrotic tissue within a burn wound. These results allowed the team to perform the first *in vivo* animal experiments in collaboration with Dr. Rodney Chan’s team at the US Army Institute for Surgical Research (USAISR) in San Antonio, Texas. The excellent porcine burn/graft model developed by the Chan lab at Brooke Army/San Antonio Medical Center^18^ led to promising results, showing the bandage ability to map the oxygenation across burns and graft sites.

Moving forward, the team was able to successfully demonstrate the bandage ability to detect static tissue ischemia, using a rat ischemic injury model provided by Dr. Samuel Lin team in the Department of Plastic and Reconstructive Surgery at the Beth Israel Deaconess Medical Center (BIDMC). Although porcine models are usually preferred for dermatological studies due to the high structural similarity between pig and human skin, using rodent models allowed for more rapid testing in a simpler, less expensive system. These three sets of experiments led to the first publication in Biomedical Optics Express,^8^ which became the journal’s most downloaded paper of the month and was highlighted by multiple media outlets including *Scientific American* and CBS News.^3^

The SPIE Photonics West Translational Research SymposiumAward was presented to Dr. Zongxi Li in Evans lab, in recognition of a translational research project and its promising first results.

**Fig. 5:**
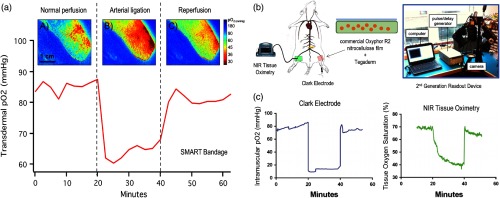
Example of transdermal ${\mathrm{pO}}_{2}$ measurements in rat lower limbs provided by the SMART bandage using an *in vivo* ischemic-reperfusion model. Results are compared with Clark electrode and NIR tissue oximetry measurements. (a) Transdermal ${\mathrm{pO}}_{2}$ maps and time-lapse measurements by the oxygen-sensing SMART bandage. (b) Experimental setup for the *in vivo* rat ischemia-reperfusion studies. (c) Intramuscular ${\mathrm{pO}}_{2}$ measured by a needle Clark electrode and tissue oxygen saturation measured by NIR tissue oximetry.^19^

Continuing the collaboration with the Lin group at BIDMC, the team used the same rat ischemic-injury model and demonstrated the bandage ability to dynamically track tissue oxygen concentration in real time during ischemic injury and reperfusion (Fig. 5). This work was published in the journal *Plastic and Reconstructive Surgery* in 2017.^19^ The paper was featured in a “Hot Topics” video presented by Dr. Rod Rohrich, editor-in-chief of the journal *Plastic and Reconstructive Surgery*.

To expand the range of potential uses, the team, in collaboration with Dr. Christene Huang at MGH, demonstrated that the SMART bandage could be used to detect tissue inflammation using complete Freund’s adjuvant induced inflammation model in a porcine model. This work was later published in Biomedical Optics Express in 2017.^20^

### 2.5 Synthesis and Scale-Up of New Ultrabright Oxygen-Sensing Phosphor (2015)

While performing the animal experiments described above, the team recognized that the commercially available Oxyphor R2, like other mesosubstituted porphyrins, was weakly absorbing and emitting, making its use challenging in normally well-illuminated clinical environments. Leveraging the Evans lab expertise in synthesizing biocompatible porphyrins for hypoxic tumor imaging,^21^ the team was able to develop new porphyrin molecules that offered an order of magnitude or more improvements in optical performance, exhibiting oxygen-dependent phosphorescence as bright as some fluorophores. The synthetic method for these new molecules along with repeated *ex vivo* experiments in the initial porcine burn explant model described above were subsequently published in Angewandte Chemie International Edition in 2015.^22^

Although the optical properties of these new phosphors were outstanding, several key steps in the synthesis suffered from low yields (micrograms) and the amount of final product generated was not sufficient to pursue further experimental work. After several attempts to solve this issue, the team was able to dramatically improve several reaction steps, which led to a robust protocol generating the molecules with higher yields (milligrams). Moreover, the scaled-up synthetic approach was designed to cleanly yield a single final product, making them Good Manufacturing Practices compatible for future steps. Subcontracting the synthesis of the precursor to a commercial partner allowed for more rapid production of these compounds in even larger quantities (grams). Importantly, these synthetic efforts set a path forward for large-scale synthesis (kilograms) to support future translational applications. Liquid bandages created with the new porphyrin molecule emitted light easily visible by the naked eye even in sunlit rooms.

### 2.6 Third-Generation Readout Device (2016)

Given the brightness of the new sensing film, the bulky, second-generation readout device was replaced by a commercial DSLR camera. In this third-generation readout device [Fig. 4(c)], the Thorlabs CMOS camera was replaced by the consumer Nikon D70s DSLR camera with the IR filter removed. In addition, modifying the emission filter setup by including a sliding filter changer allowed for faster switching between the red and green passing filters. This marked an important milestone for the clinical development of the SMART bandage, as simple and familiar cameras allowed experiments to be carried out by physicians, nurses, and technicians with minimal to no training.

### 2.7 Preparation for First-in-Human Studies (2016)

With a bright, easy-to-use bandage developed and validated in burn, grafts, ischemia and inflammation animal models, the team was prepared to enter an important phase of the translational process: first-in-human studies.

To identify a priority list of clinical applications to pursue, the team organized two clinical brainstorming sessions that brought together nine clinicians from medical academic centers and private practices in the USA and Europe. The goal was to share the status of the technology and discuss SMART bandage potential to address important clinical problems in the areas of wound care, plastic, and reconstructive surgery. Two applications were ranked as most relevant: monitoring of lower limb ischemia in diabetic patients, and postoperative monitoring of grafts and flap.

To support next-step clinical studies, the team conducted safety tests to ensure that the SMART bandage was of minimal risk to human subjects. At this stage in the design process, the team decided to restrict as many elements of the bandage as possible to those already approved by the FDA. For example, the green reference fluorophore coumarin used in some animal studies was noted to have potentially carcinogenic effects and was replaced by fluorescein, an FDA approved dye for angiography injections. Importantly as the oxygen-sensing porphyrin itself was not FDA approved, a test was set up to determine if the porphyrin would leach from the bandage under study conditions. The sensing film was applied to *ex vivo* human skin for 1 h, and after the removal of the bandage, elemental analysis of these skin samples was performed using inductively coupled plasma mass spectrometry (ICP-MS) at the Harvard T. H. Chan School of Public Health. The results demonstrated no detectable phosphor in the skin samples and served as an important safety information throughout the IRB approval process.

### 2.8 Clinical Study: Flap Monitoring (2017)

Continuing a long-standing partnership with the Lin group at BIDMC, the team was able to develop a protocol using the SMART bandage for 48-h postoperative monitoring of deep inferior epigastric perforator flaps in patients undergoing breast reconstruction surgeries post-mastectomy. The study goal was to determine if the oxygen-sensing bandage was equivalent to the current standard-of-care near-infrared (NIR) oximetry device used at the hospital. The tissue oxygenation measured by the SMART bandage ($O_{2}$-sensing nitrocellulose film and third-generation imaging device) in six subjects was found to correlate well to the blood oxygen saturation values provided by the FDA-approved NIR device.

### 2.9 Clinical Study: Chronic Inflammation (2017)

Thanks to a gift from Procter & Gamble, the team, in collaboration with Dr. Yakir Levin and the Translational Clinical Research Center (TCRC) at MGH, has recently initiated a study performed in healthy volunteers to assess inflammation caused by long term sun exposure. This ongoing study aims to not only answer an important clinical question relating to sun exposure, but also provides the team with scientific insights as to the variances encountered across population because of factors, such as sex, age, and lifestyle.

The SMART bandage prototype used in this study included a PEGylated version of the oxygen-sensing porphyrin and the third-generation imaging device. The PEGylated porphyrin formulation was put through a leaching study designed to expose the nitrocellulose film to more extreme environmental conditions than carried out previously (3-day wear at 37°C, 100% humidity). Even under these demanding conditions, the new formulation showed no sign of leaching via ICP-MS instruments at Brooks Laboratories (Bothill, Washington).

### 2.10 Fourth-Generation Readout Device (2017)

Feedback from patients and clinical study staff throughout the data collection process in both clinical studies described above gave the team key insights as to which improvements to make for the next fourth-generation readout device [Fig. 4(d)].

One of the major clinical complaints was the weight and complexity of the tripod mounted D70-based camera system. To create a lighter camera system, the team moved to a new Nikon DSLR model, the D3400, which is a fraction of the weight of its predecessor with updated features, such as Bluetooth connectivity and remote-control flash triggers.

Removal of the built-in IR filter, a cumbersome and time-consuming task, was no longer necessary thanks to a brighter sensing film and the switch to Platinum containing porphyrin that shifted the red emission to ∼640  nm. These two changes allowing the DSLR camera to be used “off-the-shelf” without any modifications. Image collection times were also reduced by replacing the two-filter switching setup with either a dual-bandpass emission filter or longpass filter. This, combined with programmable ring-mounted flash units, enabled rapid acquisition of multiple images. Where the D70 setup would require the acquisition of up to six images (e.g., green, red, and background) for a single oxygen measurement, the new system could capture all required information in a single frame in the click of a button. Though seemingly a simple change, this new configuration reduced collection times from minutes to fractions of a second, improving patient comfort, and saving a significant amount of time for both the clinician in terms of data collection, and for the research team, in terms of postprocessing image registration. This resulting fourth-generation readout device was now light enough to be handheld and simple enough to be used by clinical staff with minimal training.

### 2.11 Clinical Study: Acute Inflammation (2017)

Thanks to the collaborations with Drs. Daniela Kroshinsky and Adam Raff at MGH, the team joined an ongoing clinical study aiming to assess several technologies such as thermal imaging or reflectance spectroscopy that combined could help diagnose cellulitis, a bacteria-induced inflammatory response that is difficult to distinguish from other skin diseases due to similar clinical presentations. The SMART bandage composed of PEGylated porphyrin nitrocellulose film and fourth-generation readout device is currently being tested for the detection of the acute inflammatory component of the disease.

### 2.12 All-in-One Integrated Wearable (2018 and into the Future)

Recently, the team began to develop a miniaturized and wearable prototype combing the sensing and readout components into a single, all-in-one battery-powered unit. The prototype, taking the form factor of a smart watch or band, contains a microprocessor, a Wi-Fi chip, and a programmable controller that runs an LED and color sensor. Once again, the oxygen-sensing phosphors were chemically modified for incorporation into new solid film matrices that serve as insertable measurement “pucks.” The wearable prototypes are able to interpret oxygen concentration by comparing the detected color of the sensor emission to a look-up table, serving as a potential Internet of Things (IoT) solution that provides connected health information. Importantly, these prototype designs can be easily integrated in the future with other wearable tools for monitoring physiological parameters such as ${\mathrm{StO}}_{2}$, heart rate, skin temperature, and hydration.

### 2.13 $O_{2}$-Sensing Drug-Releasing Hydrogel Prototype (2018 and into the Future)

The SPIE Franz Hillenkamp Award in 2018 to Dr. Haley Marks in the Evans team marked an important milestone for the project and is serving an important function in driving new efforts on the drug release component of the SMART bandage technology platform. A disproportionately large fraction of the total expense associated with both acute and chronic wound care comes from oral and topical prescription drugs, with the remaining spent on screening and medical personnel time, with opportunity costs exceeding $1 billion annually.^23^ By adding the drug releasing component to the SMART bandage technology, the team aims to develop a color-changing dressing designed for open wounds that quantitatively and visually alerts clinicians of the tissue’s physiologic state. This could reduce unnecessary discomfort to patients, minimize the time and materials spent on redressing, and potentially mediate drug delivery in response to the wound bed environment.

Prior studies in the Evans team led to the development of proof-of-concept PLGA nanoparticles whose degradation could be triggered with light using photosensitizers.^24^ Recent research efforts have been focused on embedding such particles into hydrogels,^25^^,^^26^ to build oxygen sensing, drug-releasing materials. By incorporating oxygen-sensing phosphors into a hydrogel burn dressing developed by the Grinstaff lab at Boston University,^27^^–^^29^ oxygenation measurements can now be performed on complex wound topologies in transudating/exudating wound beds. Efforts focused on incorporating drug-loaded nanoparticles into this dressing formulation will enable on-demand drug release in the near-future.

## 3 Discussion and Future Perspective

It takes time and a village to move bright research ideas from fundamental research to clinical proof. The SMART bandage technology is a relevant example that illustrates this motto in the field of biomedical optics and biophotonics.

Over the last 6 years, going from the idea stage to first-in-human studies required vision, perseverance, unusual sources of seed funding and strong desire of the team to work not only across multiple scientific disciplines but also integrate the business development and commercialization principles underlying the translational process. The many accomplishments described in Fig. 2 were possible due to following: first, the research planning has been guided from the beginning by the clinical problem to be solved; second, the team included scientists and engineers with expertise in chemistry, physics, biology, and biomedical optics along with translational scientists who understood the science as well as the business and management principles involved in a successful transfer to market and patient care; third, the team worked hard to build trust and good working relations across different departments and offices at the institution level, such as the technology transfer office (Partners HealthCare Innovation) and the translational clinical research center (TCRC); fourth, establishing collaborations with other laboratories, clinicians, biotechnology companies, pharmaceutical and consumer companies, and medical device manufacturers has been an integral part of the team’s approach, and has guided the development of prototypes at each stage. Lastly and very importantly, funding from the Military Medical Photonics Program (MMPP) administered by the Air Force Office for Scientific Research, recognized the high risk/high reward nature of the research, and provided the ideal seed funding to start the project. The MMPP has a long and rich history of kick starting many innovative technologies in biomedical optics and biophotonics now currently used in clinical practice.^30^

Throughout the development process, the main technical challenges revolved around three themes: (1) creating bright oxygen-sensing phosphors that provide high signal-to-noise in standard clinical environments, (2) a biocompatible dressing formulation that can incorporate these phosphors, and (3) constructing a portable optical readout device that can be used by clinical staff with minimal training. To overcome these challenges, the team took several approaches including redesigning the core oxygen-sensing phosphor, developing a robust synthetic protocol for the large-scale production, eliminating the need for time-delay electronics, computer-control and fixed camera mounts, and reducing the number of photos required to generate oxygenation images.

The success of the project depended on determining a well-articulated set of unmet needs that served as development and engineering goals. In realizing these needs, the team chose to create a modular technology development pathway that enabled parallel and synergistic development of all components of the SMART bandage: the oxygen-sensing phosphor, sensing film, and the readout device. Moreover, by thinking about regulatory challenges early in the development process, the team was able to design their system around FDA approved materials and create formulations compatible with cGMP manufactured agents. Finally and most importantly, the team remained connected to its clinical partners, enabling continuous feedback from physicians, nurses, and caregivers throughout the development process.

Generating new intellectual property has been a major objective of the team so that commercial partners can develop and transfer the SMART bandage products to market and to patient care. Identifying the unique aspects of the SMART bandage technology against the existing landscape of patents and prior art has been and will continue to be a challenge. The fields of oxygen sensing, porphyrin chemistry, and bandages are old and broad, making straightforward assessment of the specific contribution of the team in terms of patent filing difficult. The team was able to overcome very well covered prior art in oxygen sensing with porphyrins by introducing a new concept of an integrated bandage that senses, monitors, and also releases drugs. This unique idea led to the filing in 2013 of the first method patent titled “System and methods for monitoring and treating a surface of a subject,” granted in 2017. A second composition of matter patent titled “Compounds, systems and methods for monitoring and treating a surface of a subject” was filed in 2014 to protect the sensor molecule itself and was granted in 2018. The third patent titled “System and Method for Photoluminescence Detection” was filed in 2015 to cover the time-delay detection method used for lifetime imaging of the sensing film and is still pending. A fourth pending application focuses on the wearable device and teaches methods of measuring oxygen with an integrated sensor. Overall, the team succeeded in building a solid intellectual property portfolio spanning from the system to the composition of matter and readout tool.

While several critical science milestones in the translational process have been achieved and solid intellectual property has been secured, many challenges ahead remain. The team now needs to engage in the development of an actionable go-to-market strategy for the SMART bandage, which requires an unusual set of competencies and resources for an academic institution. In addition to science, expertise, and resources in intellectual property, market analysis, regulatory strategy, and health economics need to be fully integrated to future research and development planning.^31^ This phase is critical to guide the team regarding first product to be developed. For example, a tool that monitors lower limb ischemia in diabetic patients could be a relevant application. Also, as the technology provides noninvasive real-time monitoring of limb oxygenation it has the potential to replace the point-measurement transcutaneous oxygen measurement (${\mathrm{TcPO}}_{2}$) devices currently used for assessing diabetic patients. A tool for measuring the graft/flap perfusion following reconstructive surgeries would be also an application of interest. Sports medicine applications and tools for soldiers and miners working under low-oxygen conditions are additional potential markets. Developing a go-to-market strategy will also provide the team with the rationale to understand and select the optimal exit strategy for the SMART bandage, i.e., start-up creation or license to industry.

The team continues to engage in ongoing discussions with a number of potential commercial partners and investors and is actively looking for new partners for the SMART sensing technology. We seek new academic, clinical, commercial, industrial, and venture partners who not only have key applications for the technology in mind, but also bring their own expertise to the table, so that together we can overcome clinical challenges and improve patient health.
